# Assessing the practice of written referrals to neuroradiology and how this process can be improved and standardised

**DOI:** 10.1192/bjo.2021.492

**Published:** 2021-06-18

**Authors:** Fraser Currie, Rashi Negi, Hari Shanmugaratnam

**Affiliations:** 1 University Hospitals of North Midlands; 2 Midlands Partnership Foundation Trust

## Abstract

**Aims:**

This quality improvement project aims to improve the quality of information provided in the referrals from the older adult psychiatry department to radiology when requesting neuroradiological imaging.

The secondary outcome aims to standardise information on the referral proforma. We hypothesise that this improved referral proforma will lead to improved quality of reporting from the radiology department, which will form the second stage of this quality improvement project.

A further area of interest of this exercise is to establish whether standardised radiological scoring systems are requested in the referral, as these can be utilised as a means to standardise reported information.

**Method:**

Retrospective electronic case analysis was performed on 50 consecutive radiology referrals for a period of 3 months from November 2019 to January 2020. Data were obtained from generic MRI and CT referral proforma and entered into a specifically designed data collection tool. Recorded were patient demographics, provisional diagnosis, modality of imaging, use of ACE-III cognitive score, radiological scoring systems, and inclusion and exclusion criteria.

**Result:**

Results from 50 referrals have shown: 60% were male, 40% female. Average patient age of 74, ranging from 49 to 95. 58% were referred for CT head with 42% for MRI head. More than half of referrals quoted the ACE-III score. 26% of referrals stated exclusion criteria such as space occupying lesions, haemorrhages or infarcts. 10% of referrals requested specific neuro-radiological scoring scales. Specific scales which were requested included GCA (global cortical atrophy), MTA scale (medial temporal atrophy), Koedam scale (evidence of parietal atrophy) and Fazekas (evidence of vascular changes). Only 80% of referrals included the patients GP details on the referral form.

**Conclusion:**

1. This quality improvement initiative has highlighted that the current level of information in referring patient to radiology is variable and dependent on the referrer.

2. All referrals should state exclusion criteria as per the NICE guidelines on neuroimaging in diagnosis of dementia.

3. Preliminary evidence suggests that requesting specific radiological rating scales could improve the quality of information received in the imaging report. The second part of this quality improvement initiative will aim to explore the impact of requesting these scales routinely.

